# Complete Genome Sequence of *Erwinia amylovora* Bacteriophage vB_EamM_Ea35-70

**DOI:** 10.1128/genomeA.00413-14

**Published:** 2014-08-21

**Authors:** Abdelbaset I. Yagubi, Alan J. Castle, Andrew M. Kropinski, Travis W. Banks, Antonet M. Svircev

**Affiliations:** a Department of Biological Sciences, Brock University, St. Catharines, Ontario, Canada; b Laboratory for Foodborne Zoonoses, Public Health Agency of Canada, Guelph, Ontario, Canada; c Department of Molecular and Cellular Biology, University of Guelph, Guelph, Ontario, Canada; d Al-Jabal Al-Gharbi University, Gherian, Libya; e Vineland Research and Innovation Centre, Vineland Station, Ontario, Canada; f Agriculture and Agri-Food Canada, Vineland Station, Ontario, Canada

## Abstract

The complete genome of an *Erwinia amylovora* bacteriophage, vB_EamM_Ea35-70 (Ea35-70), is 271,084 bp, encodes 318 putative proteins, and contains one tRNA. Comparative analysis with other *Myoviridae* genomes suggests that Ea35-70 is related to the *Phikzlikevirus* genus within the family *Myoviridae*, since 26% of Ea35-70 proteins share homology to proteins in *Pseudomonas* phage φKZ.

## GENOME ANNOUNCEMENT

Bacteriophages and their carrier bacterium, *Pantoea agglomerans*, have been developed as a combined biological control agent for the fire blight pathogen, *Erwinia amylovora* (1–4). *E. amylovora* phage vB_EamM_Ea35-70 will be incorporated in the phage mixture–carrier system that is applied onto open blossoms during the spring. Multiyear field-based trials have demonstrated that phages and the phage carrier bacterium *P. agglomerans* can control *E. amylovora* at efficacies comparable to those of antibiotics (1). Knowledge of phage genome structure is critical to our understanding of the biological relationship between phages and their bacterial hosts and of phage detection in the field.

The complete genome sequence of Ea35-70, a member of the family *Myoviridae*, is presented here. Ea35-70 was isolated from soil samples collected under infected pear trees in southern Ontario, Canada (4). The *Myoviridae* isolates in our collection produce clear plaques and have high titers on bacterial host cells with low exopolysaccharide production (5), while in contrast, this phage produces clear plaques on *E. amylovora* cells that produce high amounts of exopolysaccharides.

The genomic DNA of Ea35-70 was extracted and purified with the Norgen phage DNA isolation kit (Norgen Biotek Corp., Ontario, Canada).

The genome sequence was obtained from McGill University and the Génome Québec Innovation Centre (Montreal, Quebec, Canada). The sequencing library was constructed using an Illumina Nextera XL kit. The average insert size was 800 bp. The library was sequenced on an Illumina MiSeq generating 2 × 150 paired-end reads. The ends were determined by the assembly algorithm as the initiation and termination points of the De Bruijn graph. The genome was assembled using SeqMan NGen 3 (DNAStar, Madison, WI, USA). Contigs >1 kb were exported, reassembled, and proofread using SeqMan Pro (DNAStar). The final contig was oriented in the same manner as for *Pseudomonas* phage φKZ since it is the nearest genetically related phage in the database. The genomes were autoannotated using MyRAST (6), with the massaged GenBank flatfile (*.gbk) imported into Kodon (Applied Maths, Austin, TX, USA), and the annotation quality was proofread. The presence of one tRNA was identified using tRNAscan-SE 1.21 (7). The annotated sequence was searched for missed coding sequences (CDSs) and incorrect initiation codons. To determine the level of relatedness to other phages, tBLASTx, with default settings, was used to compare the predicted protein sequences to the genome sequences of all other phages in NCBI.

The genome of Ea35-70 is 271,084 bp long, with a G+C content of 49.9%. The genome contains 318 CDSs, of which 284 CDSs were annotated as encoding hypothetical proteins. Only 34 open reading frames (ORFs) were annotated as functional genes encoding structural proteins, proteins involved in nucleic acid modification, and those involved in replication.

### Nucleotide sequence accession number.

The complete genome sequence of *E. amylovora* phage Ea35-70 has been submitted to GenBank and assigned the accession no. KF806589.
